# Effects of yoga on exercise capacity in patients with lymphangioleiomyomatosis: a nonrandomized controlled study

**DOI:** 10.1186/s13023-020-1344-6

**Published:** 2020-03-16

**Authors:** Xiangfeng Li, Wenshuai Xu, Lu Zhang, Yi Zu, Yu Li, Yanli Yang, Ying Xiang, Yun Xiang, Ling Chen, Wei Liu, Lixia Chen, Kai-Feng Xu

**Affiliations:** 1 Department of Rheumatology, Peking Union Medical College Hospital, Chinese Academy of Medical Sciences, Peking Union Medical College, Beijing, 100730 China; 2 Department of Pulmonary and Critical Care Medicine, Peking Union Medical College Hospital, Chinese Academy of Medical Sciences, Peking Union Medical College, Beijing, 100730 China; 3 Department of Rehabilitation, Peking Union Medical College Hospital, Chinese Academy of Medical Sciences, Peking Union Medical College, Beijing, 100730 China; 4 Beijing Hexin Sunshine Sports Culture Co. Ltd, Beijing, China; 5 LAM Yoga Project, Beijing, China; 6 Department of International Medical Service, Peking Union Medical College Hospital, Chinese Academy of Medical Sciences, Peking Union Medical College, Beijing, 100730 China; 7 Rare Diseases Research Center, Chinese Academy of Medical Sciences, Beijing, China

**Keywords:** Lymphangioleiomyomatosis, Pulmonary rehabilitation, Yoga, Exercise capacity

## Abstract

**Objective:**

To evaluate the effects of yoga on exercise capacity and quality of life in patients with lymphangioleiomyomatosis (LAM), a rare cystic lung disease in women.

**Patients and methods:**

This was a nonrandomized, controlled study conducted in Beijing, China (August 27, 2017 – April 26, 2018). Twenty-six participants were allocated to the intervention (yoga) group (*n* = 13) or control group (n = 13). The yoga intervention involved a 24-week program of yoga class training for 90 min once a week and no fewer than 2 at-home sessions per week (at least 15 min per session). The 6-min walking distance (6MWD), lung function, serum vascular endothelial growth factor-D (VEGF-D) levels, quality of life, and symptoms of anxiety and depression were measured at baseline, 12-week and 24-week follow-up. An incremental cardiopulmonary exercise test was conducted at baseline and the 24-week follow-up.

**Results:**

Eleven patients completed the yoga training program. The yoga group exhibited improvements in the following outcomes versus those of the control group: 6MWD (+ 55 ± 29 m vs + 18 ± 49 m, *P* = 0.04), anaerobic threshold (3.4 ± 2.4 ml/min/kg vs 1.6 ± 1.4 ml/min/kg, *P* = 0.035) and peak work load (11.7 ± 14.6 W vs 0.2 ± 9.1 W, *P* = 0.027). There was no significant difference in peak oxygen consumption (VO_2_peak), lung function, VEGF-D level, and quality of life between the yoga and control groups. No adverse effects were found in the yoga group.

**Conclusion:**

Yoga is a feasible and safe intervention for pulmonary rehabilitation and potentially improves exercise capacity in patients with LAM.

**Trial registration:**

(Clinical trial registration number at www.chictr.org.cn: ChiCTR-OON-1701274)

## Background

Lymphangioleiomyomatosis (LAM) is a rare disease that typically occurs in females and is characterized as diffuse pulmonary cystic changes [1]. The symptoms include progressive dyspnea, hypoxia, recurrent pneumothorax and chylothorax. LAM can be effectively treated with sirolimus (rapamycin), which slows disease progression [2]. Most LAM patients complain of reduced exercise capacity and impaired quality of life because of airflow obstruction, abnormal diffusion capacity, dynamic hyperinflation, peripheral muscle dysfunction or pulmonary hypertension [3].

Pulmonary rehabilitation consisting of a 24-week aerobic exercise and muscle strength training significantly improved endurance time, St. George Respiratory Questionnaire (SGRQ) scores, 6-min walking distance (6MWD), dyspnea and peak oxygen consumption (VO_2_peak) [4]. Yoga is an attractive alternative option of pulmonary rehabilitation for LAM patients. The current evidence suggests that yoga exercises have beneficial effects on improvements in lung function and exercise capacity and could be used as an adjunct pulmonary rehabilitation program in patients with chronic obstructive pulmonary disease (COPD) [5]. Exertional dyspnea is an important burden associated with LAM, and many of the mechanisms are like those described in COPD. Therefore, we hypothesized that LAM patients will benefit from yoga training. In the present study, we aimed to assess the effects and safety of yoga on cardiopulmonary exercise tests, lung function, quality of life, dyspnea, anxiety and depression in LAM patients.

## Methods

### Study design and participants

The investigation was a nonrandomized, controlled, parallel study conducted in Beijing, China. All LAM patients registered in the National Rare Diseases Registry System of China (NRDRS) were evaluated for study participation. Patients had to meet the following criteria: age not less than 18 years old; living in Beijing city (for the yoga group); diagnosis of LAM according to the American Thoracic Society and Japanese Respiratory Society guidelines [6]; and clinical stability defined as no exacerbations for a minimum of 8 weeks and no change in treatment in the previous 8 weeks.

Patients were excluded for any of the following conditions: after lung transplantation or currently waiting on a lung transplantation list; pregnancy or lactation; musculoskeletal or cognitive disorders that could interfere with testing; unstable angina, respiratory tract infection within 1 month of the start of the study; previous involvement in yoga rehabilitation programs or other pulmonary rehabilitation programs within 3 months; and history of pneumothorax during the past 3 months. The protocol was approved by the Peking Union Medical College Hospital ethical committee (ZS-1398), and all patients provided written informed consent before enrolment. The study was registered at the Chinese Clinical Trial Registry (http://www.chictr.org.cn) and was assigned the following identifier: ChiCTR-OON-17012748.

The decision to perform a nonrandomized trial was made due to the rarity of the disease and geographical issues. Prior to the study, we stated that patients from Beijing would be invited to participate in the intervention, whereas patients living outside Beijing or unable to attend the yoga exercise sessions for other reasons would be invited as controls.

### Yoga intervention

Traditional hatha yoga was used in this project. The yoga intervention was carried out in a well-ventilated yoga classroom and included yoga class training once a week (90 min for each session) and home exercise of yoga that they were trained in class no fewer than 2 times a week (at least 15 min per time) during 24 weeks (from August 2017 to April 2018). The intervention was provided by certified yoga instructors who were not involved in the measurements. The curriculum was specifically designed for LAM patients based on the baseline measurement and individual physical condition. The patients were offered yoga sessions that consisted of yoga asanas (poses) interspersed with chanting and pranayama (timed breathing). The exercise was performed in a sequential, systematic and scientific way aiming at improving the overall status of the participants. The yoga participants were supposed to be able to awaken the body, know the body, and strengthen the body.

### Measurements

The primary outcome was walking distance during the 6-min walk test (6MWD). 6-min -walk test of all the patients were performed in Peking Union Medical College Hospital [7]. The secondary outcomes included VO_2_peak, anaerobic threshold (AT) during the incremental cardiopulmonary exercise test, forced expiratory volume at 1 s (FEV_1_) and forced vital capacity (FVC) measured using spirometry, daily physical activity assessed using a sports bracelet (Mambo 2, manufactured by Lifesense Medical Electronics Co., Ltd., Guangdong, China); dyspnea assessed by the modified Borg scale on a score of 0~10 on cessation of the 6-min walk test; health-related quality of life using the St. George Respiratory Questionnaire (SGRQ); symptoms of anxiety and depression using the Hospital Anxiety and Depression Scale (HADS). Safety was assessed by the occurrence of adverse events, including pneumothorax and other conditions. Adherence was assessed by the percentage of yoga sessions attended and the number of exercise sessions at home (self-reported diary).

### Statistical issues

Data were analyzed using SPSS for Windows version 20.0 (IBM Corp., USA) and are reported as the mean ± SD. The unpaired *t-*test or Mann-Whitney *U*-test was used to compare continuous variables. Categorical variables were compared using *Fisher’s* exact test. The paired *t*-test was used to compare within-subject results.

For all analyses, two-sided tests and a significance level of 0.05 was used. Intention-to-treat analyses were used for data analysis.

## Results

**Fig. 1:**
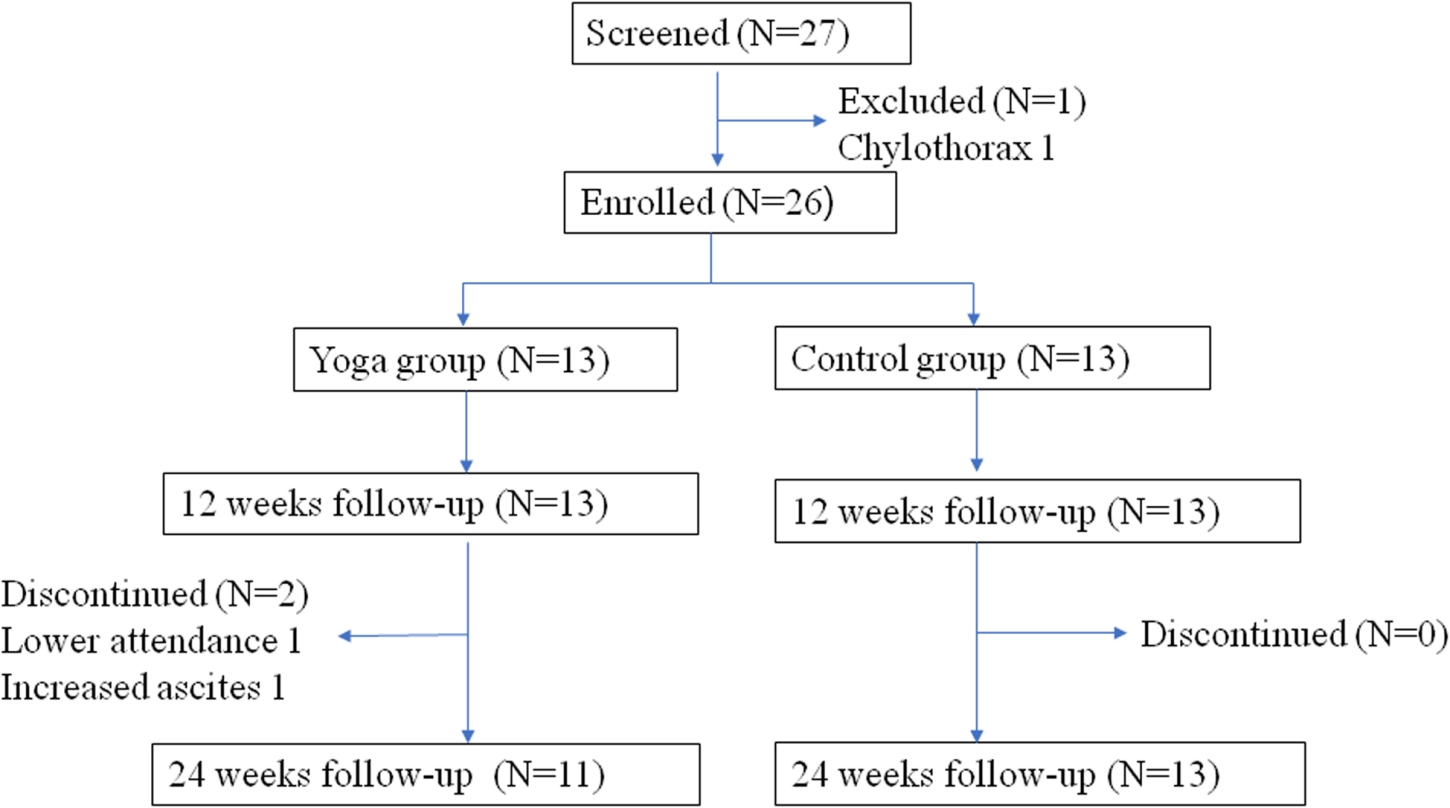
The Flowchart of the Participants

Of the LAM patients registered in the NRDRS, 13 patients each were assigned to the yoga and control groups (August 27, 2017 – April 26, 2018). Both groups were well balanced in clinical characteristics, lung function and exercise capacity (Table 1). One patient in the yoga group did not complete the program (absent from 13 sessions), and another patient in the yoga group presented increased chylous ascites. Eleven patients in the yoga group and 13 patients in the control group completed the program at 24 weeks (Fig. 1). The median attendance in the yoga group was 91.7% (IQR, 87.5, 100%). Patients did not attend the classes for reasons involving physical complaints (menstrual periods or colds) or time conflicts because of their jobs. The median home exercise duration per week was 75 min (IQR, 72.4, 104.3 min).

**Table 1:** Baseline characteristics of the participants in the two groups

Characteristics	Yoga (***N*** = 13)	Control (N = 13)	***P*** Value
Age-years	39.8 ± 8.5	43.4 ± 9.2	0.317
BMI (kg/m^2^)	21.9 ± 2.2	20.9 ± 1.5	0.201
Daily steps	9383 ± 3885	9388 ± 3962	0.998
6MWD (m)	550 ± 64	516 ± 80	0.209*†*
SpO_2_ after exercise (%)	88 ± 9	90 ± 8	0.507
Borg score after exercise	0.9 ± 1.3	1.1 ± 1.4	0.978*†*
VEGF-D, pg/ml	2957 ± 2915	2785 ± 3024	0.369*†*
**Clinical features — no. (%)**			
Sirolimus treatment	6 (46)	7 (54)	1.000*¶*
History of pneumothorax	6 (46)	3 (23)	0.411*¶*
History of angiomyolipoma	1 (8)	5 (38)	0.160*¶*
Tuberous sclerosis complex	1 (8)	1 (8)	1.000*¶*
History of chylothorax or ascites	5 (38)	3 (23)	0.673*¶*
**St. George Respiratory Questionnaire (SGRQ)**			
Symptoms	16.0 ± 13.1	15.0 ± 17.0	0.537*†*
Activity	30.1 ± 14.7	33.7 ± 25.4	0.483*†*
Impact	17.5 ± 17.6	18.2 ± 18.0	0.816*†*
Total	21.1 ± 13.1	22.5 ± 18.3	0.837*†*
**Hospital Anxiety and Depression Scale (HADS)**			
Anxiety	4.5 ± 8.8	2.9 ± 2.1	0.624*†*
Depression	1.6 ± 2.4	1.4 ± 1.6	0.830*†*
**Pulmonary function testing**			
FEV_1_ (ml)	2009 ± 639	2078 ± 845	0.816
FEV_1_%pred	72.0 ± 21.6	77.3 ± 28.0	0.594
FVC (ml)	3196 ± 628	3306 ± 481	0.621
FVC%pred	98.5 ± 16.5	105.8 ± 12.2	0.213
FEV_1_/FVC (%)	62.6 ± 14.3	62.6 ± 22.1	0.995
**Incremental CPET**			
VO_2_peak (ml/min/kg)	15.4 ± 3.3	14.4 ± 3.9	0.467
AT (ml/min/kg)	10.1 ± 1.9	9.7 ± 2.1	0.616
Peak work load (W)	88.5 ± 24.0	82.8 ± 27.5	0.574
Breathing Reserve (L/min)	41.69 ± 21.52	45.31 ± 33.32	0.511

The data are expressed as the means±SD

*Abbreviations*: *AT* anaerobic threshold, *BMI* body mass index, *CPET* cardiopulmonary exercise test, *FEV*_*1*_ forced expiratory volume in 1 s, *FVC* forced vital capacity, *SpO*_*2*_ pluse oxygen saturation, *VEGF-D* vascular endothelial growth factor-D, *VO*_*2*_*peak* peak oxygen consumption, *6MWD* 6-min walking distance

*†* The *P* value was calculated with the use of the Wilcoxon rank-sum test

*¶* The *P* value was calculated with the use of *Fisher’s* exact test

### Primary outcomes

**Fig. 2:**
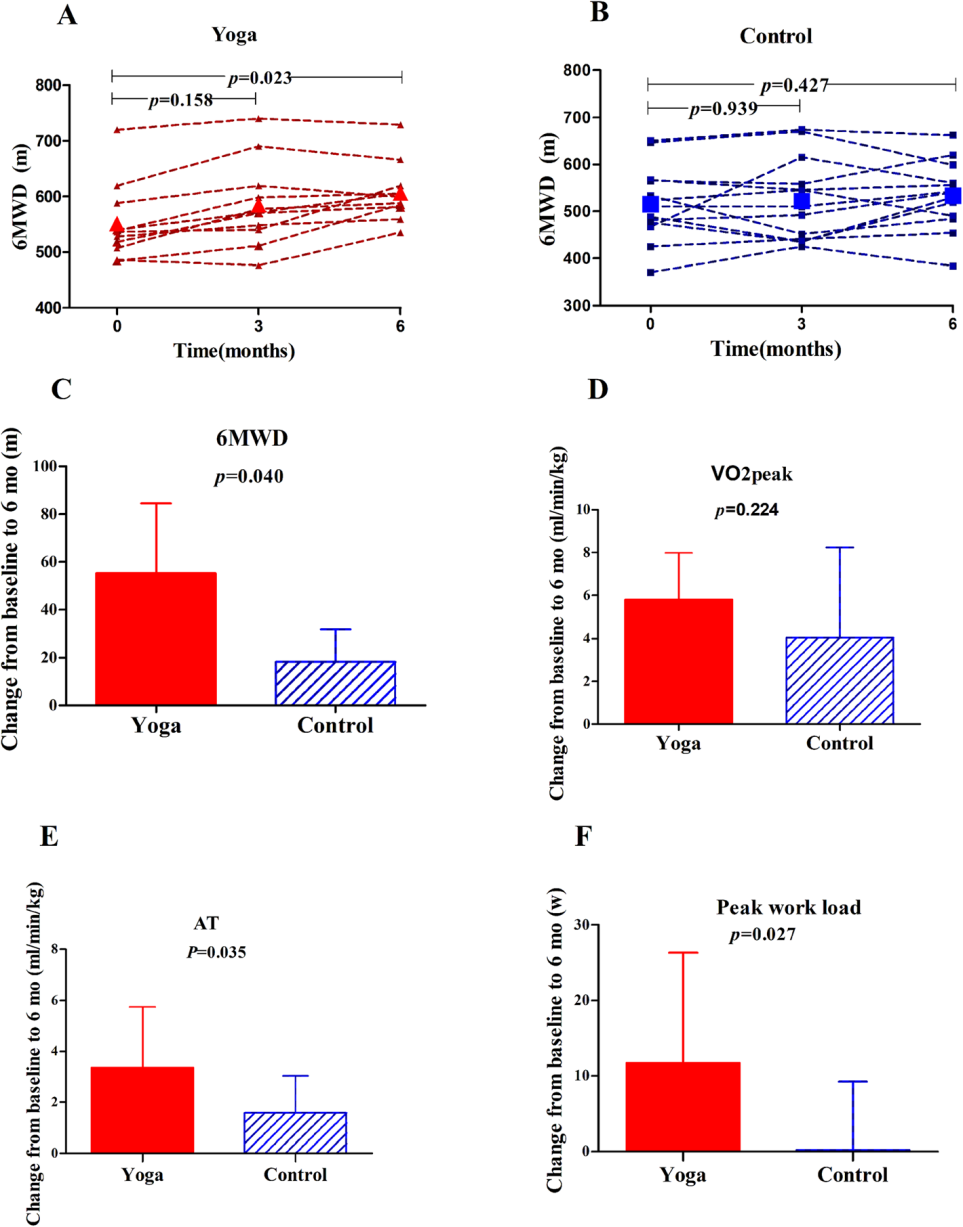
Comparison of Six Minute Walking Test and Incremental Cardiopulmonary Exercise Test between Yoga and Control Groups. Six minute walking distance (6MWD) at baseline, 3 months and 6 months in yoga group (**a**) and control group (**b**). Changes of 6MWD (**c**), peak oxygen consumption (VO_2_peak) (**d**), anaerobic threshold (AT) (**e**) and peak work load (**f**) from baseline to 6 months after yoga exercise or control

The difference in the 6MWD between the yoga and control groups was obvious over time. A significant difference was obtained at 24 weeks (*P* = 0.011) (Fig. 2). Within the groups, the increase in the 6MWD from baseline to 24 weeks was 18 ± 49 m in the control group and 55 ± 29 m in the yoga group. The post-intervention changes from baseline in the yoga group were significant (*P* = 0.04) (Fig. 2). 7/13 from control group and 9/11 from yoga group reached a 30-m increase of 6MWD. The difference using that cut-off was not significant between groups (*P =* 0.211).

### Secondary outcomes

Within each group, significant improvements emerged for VO_2_peak (the yoga group 21.2 ± 3.4 ml/kg/min vs 15.4 ± 3.5 ml/kg/min, *P* = 0.003; the control group 18.5 ± 5.5 ml/kg/min vs 14.4 ± 3.8 ml/kg/min, *P* = 0.016) and AT (the yoga group 13.1 ± 2.7 ml/kg/min vs 9.8 ± 2.2 ml/kg/min, *P* = 0.030; the control group 11.3 ± 2.4 ml/kg/min vs 9.7 ± 2.1 ml/kg/min, *P* = 0.001). After a 24-week intervention, the AT increased more in the yoga group than in the control group (3.4 ± 2.4 ml/kg/min vs 1.6 ± 1.4 ml/kg/min, *P* = 0.035) (Fig. 2). However, in comparisons between the groups, no difference was found in the VO_2_peak change (5.8 ± 2.2 ml/kg/min vs 4.1 ± 4.2  ml/kg/min, *P* = 0.224) (Fig. 2). In addition, the peak work load in the incremental cardiopulmonary exercise test increased more in the yoga group than in the control group (11.7 ± 14.6 W vs 0.2 ± 9.1 W, *P* = 0.027) (Table 2) (Fig. 2).

**Table 2:** Effects of yoga on outcome variables

Variable	Value at 6 months			Change from baseline to 6 months		
	Yoga (***N*** = 11)	Control (***N*** = 13)	***P*** Value	Yoga (N = 11)	Control (***N*** = 13)	***P*** Value
Daily steps	9305 ± 3010	8042 ± 4431	0.432	− 912 ± 2229	− 1346 ± 1416	0.585
6MWD (m)	606 ± 53	534 ± 72	0.011	55 ± 29	18 ± 49	0.040
SpO_2_ after exercise	89 ± 9	89 ± 9	0.903	0.5 ± 8.2	−1.5 ± 3.1	0.058*†*
Borg score after exercise	0.4 ± 0.8	0.6 ± 0.8	0.267*†*	−0.4 ± 0.8	−0.5 ± 1.1	0.737
VEGF-D, pg/ml	2079 ± 1149	2913 ± 2874	0.977*†*	− 183 ± 660	128 ± 654	0.505*†*
**St. George Respiratory Questionnaire (SGRQ)**						
Symptoms	9.6 ± 10.5	12.5 ± 13.0	0.681*†*	−4.6 ± 8.2	−2.5 ± 12.6	0.416*†*
Activity	26.5 ± 13.5	31.2 ± 22.0	0.485*†*	−2.4 ± 11.6	−2.5 ± 7.8	0.882*†*
Impact	8.8 ± 5.2	14.5 ± 14.0	0.640*†*	−3.0 ± 10.2	−3.7 ± 10.1	0.769*†*
Total	15.5 ± 6.1	19.3 ± 15.0	0.384*†*	−1.9 ± 7.5	−3.2 ± 6.5	0.682*†*
**Hospital Anxiety and Depression Scale (HADS)**						
Anxiety	0.9 ± 1.4	1.8 ± 2.1	0.283*†*	−0.8 ± 1.5	−1.1 ± 1.8	0.632*†*
Depression	0.7 ± 0.8	0.8 ± 1.2	0.678*†*	−0.5 ± 2.3	−0.6 ± 1.1	0.197*†*
**Pulmonary function testing**						
FEV1 (ml)	2062 ± 612	1974 ± 770	0.763	−29 ± 182	−104 ± 143	0.310*†*
FEV1%pred	74.8 ± 19.8	74.3 ± 25.2	0.957	−0.8 ± 6.6	−3.0 ± 5.5	0.432*†*
FVC (ml)	3252 ± 606	3225 ± 457	0.898	9 ± 268	−82 ± 149	0.581*†*
FVC%pred	101.6 ± 14.5	105.5 ± 14.0	0.511	0.8 ± 8.8	−0.2 ± 4.9	0.681*†*
FEV1/FVC	62.8 ± 12.7	61.1 ± 20.6	0.803	−1.2 ± 4.2	5.3 ± 23.8	0.750*†*
**Incremental CPET**						
VO_2_Peak (ml/min/kg)	21.2 ± 3.4	18.5 ± 5.5	0.164	5.8 ± 2.2	4.1 ± 4.2	0.224
AT (ml/min/kg)	13.1 ± 2.7	11.3 ± 2.4	0.089	3.4 ± 2.4	1.6 ± 1.4	0.035
Peak work load (W)	103.1 ± 23.5	82.9 ± 27.6	0.070	11.7 ± 14.6	0.2 ± 9.1	0.027
Breathing Reserve	40.36 ± 21.25	46.46 ± 29.47	0.608	−3.27 ± 8.91	1.15 ± 9.63	0.186

The data are expressed as the means±SD

*†* The *p* value was calculated with the use of the Wilcoxon rank-sum test

Abbreviations: see Table 1

There was no significant difference between the two groups with respect to the FEV_1_ or the FVC change from baseline to 24 weeks (Table 2).

There was no significant difference in anxiety and depression levels between the yoga group and the control group at baseline and 24 weeks, with adjustment for baseline levels of variables (*P* = 0.632, 0.197).

There was no significant difference in the SGRQ slope between the two groups.

No pneumothorax, yoga-related injuries or other major adverse events were reported during the study.

## Discussion

Our study showed that yoga for 24 weeks had beneficial effects in patients with LAM, especially for exercise tolerance. The main finding of this study was the significant improvement in the 6MWD and AT in the yoga group. No effects on VO_2peak_, pulmonary function, dyspnea, anxiety and depression symptoms, or quality of life scores were observed. In addition, the yoga pattern was not difficult to achieve and maintain and was well tolerated by patients. The patients were adherent to the classes and able to safely participate after the 24-week program.

Pulmonary rehabilitation is a standard practice that includes exercise training, education and behavior modification for patients with chronic lung diseases [8]. Such therapy can reduce dyspnea, increase exercise capacity, and improve quality of life. A controlled clinical trial consisting of 40 patients with LAM examined the effects of pulmonary rehabilitation [4]. The pulmonary rehabilitation group exhibited improved exercise endurance time, quality of life, 6MWD, and peak oxygen consumption. These results suggest that a pulmonary rehabilitation program should be employed in dyspneic patients with LAM. Despite compelling evidence for the benefit of pulmonary rehabilitation, only a very small percentage of eligible people attend a program. There are established barriers against enrollment and participation in center-based pulmonary rehabilitation programs related to referral practices, travel, transportation, disability and lack of program staffing [9, 10]. Flexibility of site selection in yoga training makes it easy to adhere in the program.

Yoga originated in ancient India and may denote the union between the individual self and the transcendental self. It has been included as a component of exercises prescribed for many pulmonary rehabilitation programs. The body organs and systems are cleansed through asanas (postures) and pranayama (controlling the breath). Yoga breathing or pranayama is beneficial for COPD. Previous research indicates that yoga is safe and feasible in participants with COPD [11]. Additionally, yoga participants with COPD have greater improvements in symptoms and functional performance.

In the present study, the 6MWD increase in the yoga group after 24 weeks was 55 ± 29 m. We observed a moderate increase in the 6MWD after yoga exercise; this increase is considered clinically relevant and superior to the variation obtained in a meta-analysis of COPD patients [5], which reported a mean difference of 39 m in the 6MWD after yoga training. We believe that yoga training has a positive effect on improving the exercise capacity of patients with LAM. When comparing groups using the generally accepted as clinically relevant cut-off for 6MWD of 30 m, the difference found was not significant. However, both groups did have a relatively preserved walked distance in baseline test different from what is observed in other respiratory diseases. Considering that particularity a cut-off point of 60-m increase might be better suited to compare groups which indeed showed a statistically significant difference favoring the intervention group (data not shown).

Though the peak oxygen consumption did not increase significantly in comparison to the control group, the improved AT might suggest an improvement in the aerobic capacity of the yoga group. These same results have been observed in COPD patients after practicing Hatha yoga [12, 13]. The AT elevation was remarkably higher in the yoga group, suggesting a delayed onset of anaerobic metabolism and lactic acid accumulation after exercise training. When investigating the suggested mechanisms underlying the improvements observed in AT, peak work load and 6MWD, it is reasonable to consider that different factors might be implied including desensitization to dyspnea and increased muscle strength. Also, it is not possible to completely exclude a motivation bias contributing to the greater performance in the intervention group. Another article regarding LAM rehabilitation described peripheral muscle adaptation resulting in better oxidative capacity as the main mechanism implied in the improvements they found [4]. Although that effect is classically associated with aerobic exercise it is possible to speculate that it could also happen with yoga if a significant elevation in heart rate and breath response were induced by the activity. However, we did not have enough evidence to support that hypothesis. The specific mechanisms implied need to be further studied.

FEV_1_ of both the control group and yoga group showed a significant reduction. The reduction rate of FEV_1_ in the yoga group was less than that in the control group; however, there were no significant differences between the two groups. A meta-analysis of COPD patients found that yoga training improved FEV_1_ [5].

Apart from relaxing tense muscles, yoga can also alleviate mental pressure [14]. Slow, relaxed breathing should also enhance well-being and reduce anxiety. A recent systematic review of the effects of yoga on anxiety found that the literature was poor quality and the effect on anxiety was equivocal [15]. Our study did not find significant improvements in depression and anxiety scales, but this might be partially explained by the fact that patients who joined this study did not have depression and anxiety exhibiting scores at baseline.

The Yoga exercise is quite safe. No pneumothorax or other serious adverse events related to yoga exercise were reported during the study.

There are some limitations to this study. First, we used 6MWD and incremental cardiopulmonary test as main measurements. Constant work rate exercise test should be more sensitive in reflecting the effects of intervention in daily life. Second, the study involved only 24 weeks of total duration and involved a small group of patients. Third, we did not specifically test participant comprehension of pranayama practice, but we did monitor that actual practice by a review at the next training. Fourth, we tried to enhance retention and adherence by having the subjects keep diaries and by reminding them to complete their daily practice through interactions with WeChat (a Chinese mobile social media application that provides instant messaging services to smart terminals) every day. Our data showed that on average, participants in the yoga group practiced 75 min per week at home, which could have reduced the impact of the intervention. Fifth, the impossibility of randomizing the intervention group, which was based on geographical issues, raised concern that the differences may existed in two groups although both groups seemed to be balanced in baseline characteristics, lung function and exercise capacity.

## Conclusions

In conclusion, our findings demonstrated that yoga training may improve the functional exercise capacity in patients with LAM. Despite their frailty, patients diagnosed with LAM were able to safely perform yoga. Moreover, we suggest that yoga could be a useful adjunct pulmonary rehabilitation program for LAM patients. To help clarify the issue, further larger-scale trials with extended follow-up periods should be conducted to evaluate the long-term effects of yoga training in LAM patients.

## Supplementary information

**Additional file 1 **: **Appendix**. Online Supplementary information. Yoga exercise program used in this research for the participants with LAM.

## Data Availability

The dataset used in this research and analysis was available from the corresponding author.

## Abbreviations

6MWD: 6-min walking distance
AT: Anaerobic threshold
BMI: Body mass index
COPD: Chronic obstructive pulmonary disease
CPET: Cardiopulmonary exercise test
FEV_1_: Forced expiratory volume in 1 s
FVC: Forced vital capacity
HADS: Hospital Anxiety and Depression Scale
LAM: Lymphangioleiomyomatosis
NRDRS: National Rare Diseases Registry System of China
SGRQ: St. George Respiratory Questionnaire
SpO_2_: Pluse oxygen saturation
VEGF-D: Vascular endothelial growth factor-D
VO_2_peak: Peak oxygen consumption
