# Supplementary material for: Effects of yoga on exercise capacity in patients with lymphangioleiomyomatosis: a nonrandomized controlled study
Source: Orphanet J Rare Dis. 2020 Mar 16;15:72. doi: 10.1186/s13023-020-1344-6 (PMC7075042; doi:10.1186/s13023-020-1344-6)
Supplement: Supplementary file 1 — Additional file 1 : Appendix. Online Supplementary information. Yoga exercise program used in this research for the participants with LAM. [file 13023_2020_1344_MOESM1_ESM.docx]

**Yoga Exercise Program for the Participants with LAM**

**I. Traditional Hatha Yoga**

The exercise program of the phase I of the LAM project adopts the traditional Hatha yoga which is the systematic inheritance of the yoga’s essence and the wisdom extracted from the practices and experience during the thousands of years. The Hatha yoga has been scientifically proven to improve the functions in aspects of the physical, physiological and spiritual.

The program of the phase I has developed the corresponding curriculum and practice contents according to the specific participants' situations, aiming at improving their physical and mental states in a sequential, systematic and scientific way.

**II. Overall Objectives of Yoga Program of LAM Project Phase I**

1. To wake up the body, get any healthy knowledge on the body, and make the muscle organization and skeletons preliminarily aligned and restored;

2. To perceive the respiration, improve the physiological functions of the body, including the digestive system and circulatory system, etc.;

3. To establish the basic links with self, and cultivate the basic awareness, so as to understand their own basic mental state and various emotions, etc.

**III. Specific Programs**

**i. Workshop**

**a. 3-day intensive workshop**: firstly to help the yoga instructor know the participants’ physical and mental state, make them have a relatively systematic and clear basic understanding about the traditional yoga, and understand the purposes and roles of the sequential exercises, so as to establish a good foundation for the following six months of practices.

**b. Practices:** includes asanas (postures), chanting and pranayama (breathing exercises).

**ii. Regular weekly Course**

According to the results of the physical survey to the participants during the workshop, the following basic programs have been formulated:

**a. The first and second stages (the first four months)**: mainly to regulate the digestive system and circulatory system, including asanas (postures), chanting and pranayama (breathing exercises);

**b. The third stage (the last two months)**: to enhance the strength of the limbs, including asana, chanting and breathing exercises.

**Appendix**

**I. Asanas**

At the beginning of exercises, sitting in sukhasana with the spine relaxing upward, and focusing on the inhalation and exhalation for several minutes, and then chanting three Oms and relaxing with Savasana. The next is exercising. Between any two postures, there should be different resting postures as necessary.

During the six months, the first four months are the first stage and the rest two months are the second stage, with different exercise postures and different purposes during each stages, and a little different in the postures every two weeks.

**i. Asanas during the first stage**

1. Ekapada Uttanpadasasana

Lying on the back with both legs together, with the inhalation slowly raising one leg upward, holding on several breaths and falling back on the ground, and then repeating the same to another side; then relaxing in savasana;

2. Setu Bandhasana

Lying on the back with both legs together and arms on the body sides, bending legs with both heels touching both middle fingers respectively, and exhaling to slowly lift the pelvis relaxed off the ground as far as possible, with the whole spine and back relaxed, and after several breaths putting the whole spine on the ground step by step; then relaxing in savasana;

3. Parivrtta Makarasana

Lying on the back with both legs together and arms opened flat, putting the left heel between the big toe and the second toe of the right foot, and turning legs to right and head to left, with shoulders on the ground, after several breaths coming back to repeat the same to another side; then relaxing in savasana;

4. Anantasana

Lying on the right side, bending the right elbow with the right hand propping under right side of the head, stretching legs flat as far as possible with right heel touching the ground, and then raising the left leg upward and using the left hand holding the left big toe, and after several breaths coming back to repeat the same to another side; then relaxing in makarasana;

5. Ardha Navasana

In the prone position, with both legs together and arms straight forward, lifting the left leg, right arm and the head simultaneously, and after several breaths coming back to repeat the same to another side; then relaxing in makarasana;

6. Ardha Salabhasana

In the prone position, with both legs together, palms on the ground on both sides of chest and forehead on the ground, exhaling to slowly raising the left leg upward and holding on several breaths, and exhaling to come back and relaxing, then repeating the same to another side; then relaxing in makarasana;

7. Pranamasana

Sitting on the knees with buttocks on heels, forehead on the ground and both hands on both sides of the head, and exhaling to roll forward from the forehead along the central axis of the head as far as possible, holding on for several breaths, and then coming back to relax;

8. Vajrasana

Kneeling with buttocks on the heels, holding on several breaths, and then stretching left leg forward from left side and right leg forward from the right side, and then relaxing.

9. Marjaryasana

Kneeling upright, with two knees apart as the same wide as pelvic, both thighs and arms perpendicular to the ground with palms on the ground, exhaling to push the spine upward and then downward for several rounds, and then relaxing on Vajrasana;

10. Upavishta Konsana

Sitting upright with both legs apart greater than 90 degrees, putting the hands in the front of body, exhaling to bend the upper body forward, holding on for several breaths and then with inhalation coming back and exhaling to relax.

11. Parighsana

Kneeling upright, stretching the left leg to the left side with the right thigh perpendicular to the ground, inhaling to open both arms from the body sides, exhaling to bend the body to left side with left hand holding left ankle and right arm extending upward along the right ear, holding on for several breaths and with inhalation coming back, and repeating to another side; then relaxing in Vajrasana;

12. Adho mukha Svanasana

Kneeling with buttocks on the heels and forehead on the ground, stretching both arms forward apart, then using both arms and legs to push the pelvic upward as far as possible, with the spine stretching toward to the pelvis, holding on for several breaths and inhaling to come back, and repeating to another side; then relaxing in Vajrasana;

13. Tadasana

Standing with both legs and feet together, exhaling to raise both heels and arms upward meanwhile and finally putting both palms over the head, holding on for several breaths, and then exhaling to come back meanwhile.

14. Ardha Kati Cakrasana

Standing with both legs and feet together, inhaling to raise the left arm upward from left side and then exhaling to bend the body to right side, holding on for several breaths and then coming back to repeat to another side;

15. Savasana

Lying flat on the back with both legs apart widely and both arms a little away from the body, and relaxing whole body with palms facing up.

**ii. Asanas during the second stage**

1. Matsyasana (variant)

Lying on the back with both legs together, putting both arms under the body with palms downward under the buttocks, and with the inhalation slowly bending elbows to prop up upper body with the head top on the ground, holding on several breaths and coming back while exhaling; then relaxing in savasana;

2. Bhujangasana

In the prone position with both legs together and palms on the ground on both sides of chest, exhaling to roll up upper body from neck as far as possible comfortably, and after several breaths coming back to repeat the same to another side; then relaxing in makarasana;

3. Gomukhasana

Kneeling upright, with the hands propping in front of the body, crossing the right leg to left side from the front of left leg, and exhaling to put the buttocks on the ground between both heels, keeping the back upright and relaxed, palms on the soles for several breaths, and then coming back to repeat to another side;

4. Paschimottanasana

Sitting upright with both legs together, inhaling to lift arms upward from the sides, exhaling to bend forward, and finally two hands holding the big toes respectively and downing the forehead towards to knees for several breaths, then inhaling to raise up both arms and the upper body, and exhaling to come down and relax;

5. Vakrasana

Sitting upright, bending the left leg to put left ankle inside right knee, with left hand beside the body backward, inhaling to raise the right arm up and twist body to left, and exhaling to put right palm on the ground, with all fingers backward, holding on for several breaths and then with inhalation coming back to repeat to another side;

6. Vrksasana

Standing with both legs and feet together, exhaling to bend left leg and putting left foot sole on right thigh root inside, with palms together in the front of chest, holding on for several breaths, and exhaling to come down and then repeating to another side;

7. Prasarita Padottanasana

Standing with both feet apart widely and both hands holding on both sides of pelvis with thumbs backward, exhaling to push pelvis forward, inhaling to come back, and then exhaling to bend the upper body down forward, finally putting hands apart on the ground between the feet, with head top on ground between two hands, holding on for several breath, and then inhaling to come back;

8. Parivrtta Trikonasana

Standing with both feet apart widely, inhaling to lift arms up flat from sides of body, exhaling to twist body to left side and use right hand to grasp left ankle, and looking at left hand, holding on for several breaths and then exhaling to come back, and repeating to another side;

9. Virabhadrasana

Standing with palms together in the front of chest, exhaling to withdraw left leg backward widely and then bend right leg, holding on for several breaths and then inhaling to come back, and repeating to another side;

**iii. Breathing during exercises**

For all yoga postures, the important thing to firstly feel the breathing rhythm, so as to integrate breathing in the action effectively, making the body slowly relaxed and then stretched; to feel relaxation and expansion coming from inhalation and serenity, relaxation and posture deepened coming from exhalation.

**II. Chanting**

i. During the first four months, chanting the sound of ‘A’ 15 times after asana exercises;

i. During the fifth month, chanting the sound of ‘U’ 15 times after asana exercises;

i. During the sixth month, chanting the sound of ‘OM’ 15 times after asana exercises.

**III. Breathing exercise (pranayama)**

i. Sitting pose

Sukhasana adopted during breathing exercises, i.e. both legs crossing each other with the foot under the opposite leg and hands placed on knees respectively, the spine relaxing straight, and shoulders and arms relaxing, with breathing evenly all through.

ii. Breathing exercises:

1. Surya-Bhedana pranayama: Inhaling from right nostril and exhaling from left nostril, 10 rounds
2. Chandra-Bhedana pranayama: Inhaling from left nostril and exhaling from right nostril, 10 rounds

iii. Exercise instructions

1. At the initial stage, exhaling longer than inhaling, and at the end of project, the ratio of exhaling: inhaling =1:2 for those participants who are capable to do like this;

2. Ujjayi breathing method adopted, i.e., the sound of “so” made while inhaling and “ham” made while exhaling, with relaxed, even and soft breathing;

3. Abdomen kept immovable as far as possible.

4. Metronome adopted for timing;
